# Maternal Deaths in NSW (2000–2006) from Nonmedical Causes (Suicide and Trauma) in the First Year following Birth

**DOI:** 10.1155/2013/623743

**Published:** 2013-08-19

**Authors:** Charlene Thornton, Virginia Schmied, Cindy-Lee Dennis, Bryanne Barnett, Hannah Grace Dahlen

**Affiliations:** ^1^University of Western Sydney, Locked Bag 1797, Penrith South, NSW 2751, Australia; ^2^University of Toronto, 155 College Street, Toronto, ON, Canada M5T 2P8; ^3^University of New South Wales, High Street, Kensington, NSW 2050, Australia

## Abstract

*Introduction*. Trauma, including suicide, accidental injury, motor traffic accidents, and homicides, accounts for 73% of all maternal deaths (early and late) in NSW annually. Late maternal deaths are underreported and are not as well documented or acknowledged as early deaths. *Methods*. Linked population datasets from births, hospital admissions, and death registrations were analysed for the period from 1 July 2000 to 31 December 2007. *Results*. There were 552 901 births and a total of 129 maternal deaths. Of these deaths, 37 were early deaths (early MMR of 6.7/100 000) and 92 occurred late (late MMR of 16.6/100 000). Sixty-seven percent of deceased women had a mental health diagnosis and/or a mental health issue related to substance abuse noted. A notable peak in deaths appeared to occur from 9 to 12 months following birth with the odds ratio of a woman dying of nonmedical causes within 9–12 months of birth being 3.8 (95% CI 1.55–9.01) when compared to dying within the first 3 months following birth. *Conclusion*. Perinatal services are often constructed to provide short-term support. Long-term identification and support of women at particular risk of maternal death due to suicide and trauma in the first year following birth may help lower the incidence of late maternal deaths.

## 1. Introduction

The maternal mortality rate (MMR) (death within 42 days of delivery/early deaths including direct, indirect and incidental subcategories) in Australia in 2010 was 7/100,000 confinements [1]. Direct deaths are those which occur as a result of the pregnant state whilst indirect deaths are the result of preexisting conditions or new conditions exacerbated by the pregnancy but not attributable to the pregnancy. Incidental deaths are those occurring from conditions which arose during the pregnancy but for which the pregnancy is unlikely to have contributed to the death. The leading contributors to the MMR worldwide are haemorrhage, sepsis, and the hypertensive disorders of pregnancy [2]. The accurate ascertainment of these deaths was reported to be 82% of cases via standard reporting means in New South Wales (NSW), with data linkage from multiple reporting sources shown to greatly enhance the catchment of cases in numerous studies both within Australia and internationally [3–5].

The extent of late maternal deaths (those occurring >42 days after delivery and up to one year of birth) is reported to be underestimated by up to 50% in most studies conducted which utilised multiple collection means to calculate rates [3, 4, 6–8]. Late maternal deaths are not included in the standard World Health Organisation (WHO) MMR estimates but it has been reported that the relative risk (RR) of death for a woman within 91 days of birth in developing nations is 2.8 when compared to baseline reproductive years' mortality, with the RR returning to baseline at one year postpartum [9]. Late maternal deaths are also subclassified as direct, indirect, and incidental. The last category is underreported or excluded from reporting but has a significant impact on the capacity of policy makers and care providers to understand the potential social and domestic situations which make some women more vulnerable following the birth of a baby.

Trauma, including suicide, accidental injury, motor traffic accidents, and homicides, is the leading nonobstetric cause of late maternal death in the United States [10] and accounted for 73% of all maternal deaths (early and late) in NSW over an eight-year study period [3]. These nonmedical deaths, and most notably, the late maternal deaths, are underreported [11] and are not as well documented or acknowledged as the obstetric causes listed above. Factors associated with nonmedical death include intimate partner violence (IPV), which is reported to occur in 17%–27% of Australian pregnancies [12, 13], and perinatal anxiety and depression, with one in eight Australian women reporting depressive symptoms in the year following birth, and rates of diagnosed postnatal depression (PND) are reported to be 10% of Australian postnatal women [14]. Other contributing factors include mental health disorders and substance abuse [15, 16].

The aim of the study is to evaluate the rate and causes of maternal mortality up to one year following birth utilising linked data methodology to ascertain accurate rates of maternal mortality and the potential role played by nonmedical causes in these deaths.

## 2. Methods

### 2.1. Data Sources

Perinatal data recorded in the NSW Midwives Data Collection (MDC), hospital admissions recorded in the Admitted Patient Data Collection (APDC), and deaths recorded in the Registry of Births, Deaths and Marriages (RBDM) and the Australian Bureau of Statistics (ABS) were provided by NSW Department of Health for the period from 1 July 2000 to 31 December 2007. The MDC is a population-based surveillance system containing maternal and infant data on all births of greater than 400 grams birth weight or 20 weeks gestation. The APDC is a collection of records of all services to admitted patients provided by New South Wales Public Hospitals, Public Psychiatric Hospitals, Public Multi-Purpose Services, Private Hospitals, and Private Day Procedures Centres. The NSW RBDM and ABS contain mortality information for deaths occurring in NSW and Australia.

The linked datasets were provided by the NSW Centre for Health Record Linkage (CHeReL) following approval by the Data Custodian (NSW Health). Probabilistic data linkage techniques were utilised for these purposes and deidentified datasets were provided for analysis. Probabilistic record linkage software works by assigning a “linkage weight” to pairs of records. For example, records that match perfectly or nearly perfectly on first name, surname, date of birth, and address have a high linkage weight, and records that match only on date of birth have a low linkage weight. If the linkage weight is high, it is likely that the records truly match, and if the linkage weight is low it is likely that the records are not truly a match. This technique has been shown to have a false positive rate of 0.3% of records [17].

The deaths recorded in the ABS data were compared to those recorded in the NSW RBDM to enhance the identification of episodes and duplicates were removed. Those deaths which were recorded as occurring within 365 days of a birth (as noted in the MDC) were included in the datasets. Utilising the unique identifier, the APDC was then searched for admission data prior to the birth, admission during the birth, and admissions which occurred between the birth and the maternal death for associated diagnoses and co-morbidities.

Ethical approval was obtained from the NSW Population and Health Services Research Ethics Committee, Protocol no. 2010/12/291.

### 2.2. Outcome Measures

Maternal death and associated causes were categorised using the International Statistical Classification of Diseases and Related Health Problems, Tenth Revision, Australian Modification (ICD-10-AM) [18] as recorded in the NSW RBDM, ABS, and the APDC. A nonmedical death for this purpose was defined as the death of a woman within 365 days of giving birth due to means not defined by medical illness. The deaths were further divided into four categories of trauma: intentional self-harm (suicide), accidental injury, transport accidents, and homicide. Standard definitions of early (within 42 days of birth) and late (>42 to < or = 365 days) were applied for these purposes.

### 2.3. Data Analysis

Descriptive statistics (frequencies and proportions) were calculated. Demographics comparisons, events by time from birth, and risk of death by suicide were compared to the general female population. All analyses were conducted using IBM SPSS v.19.

## 3. Results

During the 6-year time period, there were 552 901 births and a total of 129 maternal deaths. Of these deaths, 37 occurred within 42-day time period following birth, equal to an early MMR of 6.7/100 000 confinements occurring following the recorded birth after 20 weeks gestation. The remaining 92 deaths occurred in the interval between 42 days and 365 days following birth, equal to a late MMR of 16.6/100 000. The demographics of all women in the cohort are summarised in Table 1 with comparisons being made between those women who died and those who did not. In summary, the deceased cohort was more likely to smoke (42.6% versus 15.3%), develop either or both gestational diabetes and/or pregnancy related hypertension (18.5% versus 9.7%), and deliver at an earlier gestation (37.0 versus 39.1 weeks) to a smaller baby (2837 versus 3403 grams) who was more likely to require admission to a neonatal intensive care unit (15.1% versus 2.2%) and more likely to have subsequently died (PMR 101/1000 versus 9/1000).

Forty-eight (37%) of all deaths were the result of nonmedical causes or trauma, four of which occurred in the early time period and 44 in the late time period. The deaths were further divided into four categories: intentional self-harm, accidental injury, transport accidents, and homicide. The reasons for death are outlined in Table 2.

Of the 48 women who died as a result of nonmedical causes, 67% (*n* = 32) had a mental health diagnosis and/or a mental health issue related to substance abuse (i.e., dependency) noted on an admission to hospital during pregnancy, birth, or in the interval between the birth and their death. The greatest percentage of such comorbidities was noted in women who died from suicide (73%, *n* = 11) or accidental injury (73%, *n* = 11).

**Figure 1:**
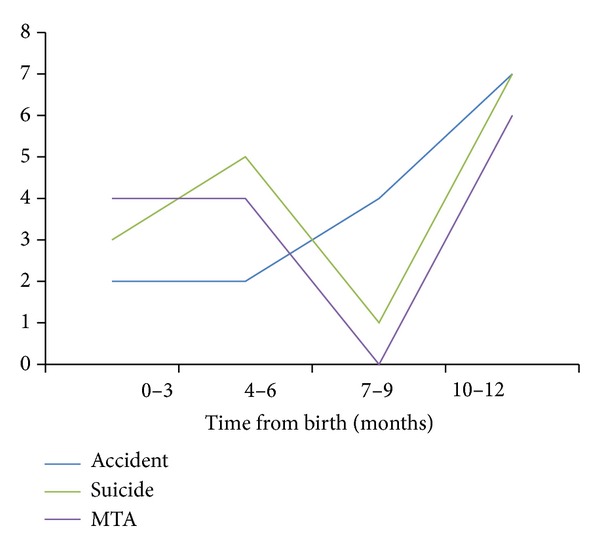
Events and time from birth in 3 monthly increments. MTA—motor traffic accident.

An examination of nonmedical or traumatic death events and time from birth was undertaken to determine if there is an increased risk time for trauma to occur. These results are shown in Figure 1. A notable peak in deaths appeared to occur from 9 to 12 months following birth with the odds ratio of a woman dying of nonmedical causes within 9–12 months of birth being 3.8 (95% CI 1.55–9.01) when compared to dying within the first 3 months following birth.

The risk of death by intentional means (suicide) in the cohort was also examined in comparison to the age-specific death rates in the Australian population with a resulting relative risk of suicide of 0.9 (95% CI; 0.29–2.59).

## 4. Discussion

### 4.1. Suicide/Accidental Injury

We have shown that suicide and accidental injury are the two leading causes of death for women within one year of birth, contributing to 62% of all causes during this period, a similar result to that shown by Cliffe et al. [3] in NSW in 2008 and in international studies [11, 19]. Suicide and accidental injury are grouped in this fashion due to the nature of the specificities of the accidental injury and the high likelihood that many deaths coded as accidental were potentially intentional in nature, including deaths by overdose/accidental poisoning, firearm discharge, and deaths of unknown cause. In this study, 73% of suicides were conducted by violent means (jumping from high place, lying in front of moving object, gunshot, and strangulation/suffocation), findings which have been reported in another study in the Australian setting [20]. Such violent methods are less common in female suicides in other age groups. The WHO study of suicide incidence and means reported that violent and highly lethal methods such as firearm suicide and hanging are more frequent among men, whereas women often choose poisoning or drowning, which are less violent and less lethal [21]. Suicide as a cause of death is also less frequent in women than men with the standardised death rate from suicide in men as 16.1/100 000 and in women as 4.4/100 000 [22].

### 4.2. Comorbidities

Mental health diagnoses and/or drug or alcohol dependence occurred in 73% of women who died as the result of intentional self-harm or accidental causes. These issues were highlighted during the pregnancy and/or during the birth admission or during postnatal admissions and were therefore known to health professionals prior to death, a finding reported by Austin. The use of screening tools such as the Edinburgh Perinatal Depression Scale (EPDS) [23] has been recommended for the detection of depressed women both antenatally and postnatally [24] with at-risk women being then directed towards appropriate mental health services [25]. This study highlights the ongoing need for identification and support of these at-risk women, with current NSW Health Guidelines recommending the screening of all pregnant women for depression with the EDPS at the initial antenatal visit [26], again at the 6–8-week visit postnatally and at the 6–8-month time point. This highlights the need for ongoing assessment of women in the first year following birth and the implementation of service provision pathways which support women and their families.

Other factors noted in the deceased cohort were the high rates of smoking during pregnancy (>2.5 times more likely to smoke) and almost double the rate of pregnancy-related medical conditions when compared to those women in the cohort who survived, conditions which may also be indicative of lower socioeconomic status within the deceased cohort. The women in the deceased cohort had smaller birth weight babies, born at shorter gestations and who were admitted to a neonatal intensive care unit at a rate more than seven times higher than the remaining cohort. The cascade of smoking during pregnancy, early birth, lower birth weights, NICU admission and perinatal mortality is displayed in the deceased cohort. The stress of these events on women has been well documented and such risk factors are seen frequently in women diagnosed with postpartum depressive symptoms [27, 28]. Combining these issues with preexisting psychiatric comorbidities including substance abuse potentially places women at severe risk of adverse outcomes. The published results of a regression analysis undertaken examining factors such as these showed that the strong factors associated with the development of postpartum depressive symptoms were a history of personal depression, being physically abused as a child, having a partner with a substance abuse issue, and poverty [29].

### 4.3. Timing

By dividing the first year after birth into three-monthly increments, it can be seen in Figure 1 that there is an increased occurrence of traumatic death in the 9–12-month period following birth. The causes may be multiple. Firstly, the proportion of women reporting depressive symptoms is higher 9 to 12 months following birth [30]. An Australian study [31] found that 69% of women at 7 months postpartum reported tiredness and 16.9% reported depressive symptoms with a cumulative effect of tiredness and an associated effect on mood and mental acuity [32]. Secondly, this is the time when many women return to the workplace, resulting in additional stress factors from work and childcare demands. Thirdly, there is a weaning of support services as the months from birth increase and a hesitancy for women to seek assistance for mental health issues or to use mental health services [33, 34]. Fourthly, for women who already have a diagnosis or are known to psychiatric services, many services within this setting cease at the 9–12-month point postpartum [35, 36], and finally, for some women the provision of services simply does not meet their individual needs due to a lack of coordination and integration among services [37]. These findings highlight the need for early identification and extended postpartum services for “at-risk” women, such as those with mental health and/or substance abuse issues.

In developed countries, women have a high expectation that the period following childbirth will be both positive and fulfilling and that the changed circumstances of motherhood will improve the overall quality of life. Women in our society are frequently shown, for example, in the media, a picture of motherhood that is not factual in nature and promotes unrealistic expectations [38]. The reality for many women is that motherhood increases dissatisfaction and induces or augments preexisting conditions or predilections to anxiety and depression [39]. The high incidence of postnatal depression (PND) in Australian women (1 in 10 pregnancies) supports this proposition [40].

The strengths of this study are the size of the dataset available for analysis, the accuracy of the linkage process, and the depth of coding which is attached to admission data. The weaknesses lie in the lack of socioeconomic and sociodemographic details available which would add a more detailed dimension of analysis to occur.

## 5. Conclusion

This study sought to examine maternal mortality up to one year following birth and to ascertain rates of maternal mortality and the role played by nonmedical causes in these deaths. During the 6-year time period, 37 deaths occurred within 42 days following birth with 92 deaths occurring in the interval between 42 and 365 days. Forty-eight of the deaths were the result of trauma, which mainly occurred after 42 days following birth. There appears to be a spike in maternal deaths in the period of nine to twelve months postpartum and this may well correspond to a reduction in perinatal services and other support or/and an increase in stressors as women are reentering the workforce. There is a strong association with women who have co-morbidities, in particular mental health and/or substance abuse. There was high rate of obstetric complications and intervention, low birth weight infants, admission of infants to NICU and perinatal mortality. Perinatal services are often constructed to provide short-term support. Long-term identification and support of women at particular risk of maternal death due to suicide and trauma in the first year following birth may help lower the incidence of late maternal deaths.

The term “perinatal” covers the period from conception to one year postpartum. Services need to remain cognisant of the needs of new mothers throughout this vulnerable period.

## Figures and Tables

**Table 1:** Demographics and birth outcomes.

	Cohort	Deceased	*P*
Age (years)	30.1 (5.57)	30.4 (6.55)	0.63
Primiparous	42%	37%	0.33
Gestation at birth (weeks)	39.1 (2.06)	37.0 (4.00)	**<0.001**
Preexisting diabetes or hypertension	1.5%	2.3%	0.45
Pregnancy related diabetes or hypertension	9.7%	18.5%	**0.002**
Smoking during pregnancy	15.3%	42.6%	**<0.001**
Normal vaginal delivery	63.5%	55.0%	**0.01**
Birthweight (grams)	3402.6 (576.91)	2836.5 (900.90)	**<0.001**
Admitted to NICU	2.2%	15.1%	**<0.001**
Perinatal mortality rate	9/1000 births	101/1000 births	**<0.001**

**Table 2:** Principal cause of death for all deaths associated with trauma.

Cause of death	%	*n*	Mental health comorbidity	Substance abuse	Mental health and/or substance abuse
Intentional self-harm (includes drowning, jumping from high place, lying in front of moving object, inhalation of vapours (carbon monoxide), hanging/strangulation/suffocation, overdose, and gunshot)	31%	15	40%	33%	73%
Accidental injury (includes drowning, firearm discharge, poisoning by alcohol or other drugs, and unspecified/unknown)	31%	15	33%	40%	73%
Transport accidents (includes driver, passenger, pedestrian, and other vehicular incidents)	29%	14	29%	7%	36%
Homicide	8%	4	0%	50%	50%
